# 1322. Outcomes Associated with Primary Antibiotic Therapy in Spinal Epidural Abscess due to *Staphylococcus aureus: C*omparison of First-line vs Alternative Agents

**DOI:** 10.1093/ofid/ofad500.1161

**Published:** 2023-11-27

**Authors:** Madeline Fowler, Jessica Phan, Rupal K Jaffa, Travis J Carlson, Leigh Ann Medaris, Julie E Williamson

**Affiliations:** Carolinas Medical Center, Charlotte, North Carolina; Alexander T. Augusta Military Medical Center, Woodbridge, Virginia; Atrium Health, Charlotte, North Carolina; High Point University Fred Wilson School of Pharmacy, High Point, North Carolina; Atrium Health, Charlotte, North Carolina; Atrium Health, Charlotte, North Carolina

## Abstract

**Background:**

Spinal epidural abscess (SEA) is an uncommon but serious infection with potentially devastating sequelae. For SEA due to methicillin- susceptible *S. aureus* (MSSA), anti-Staphylococcal penicillins have been considered first line, however retrospective data have failed to show a difference in outcomes compared to cefazolin. Comparative data in methicillin-resistant *S. aureus* (MRSA) SEA are sparse.

**Methods:**

This was a multisite, retrospective cohort of adults admitted to an acute care or acute rehabilitation hospital from 1/1/2017 – 9/10/2021 with confirmed *S. aureus* SEA by radiology and microbiology (blood and/or surgical cultures). Recurrent or polymicrobial SEA, endocarditis, meningitis, or prosthetic joint infection cases were excluded. Therapy was grouped as lower (LCNS) or higher (HCNS) CNS penetration (Fig. 1) and was assessed as overall principal therapy (OPT) (definitive therapy for > 50% of the total course) and early principal therapy (EPT) (definitive therapy for > 50% of the first 2 weeks). Patients were also analyzed by MSSA vs MRSA. The primary outcome was composite 90-day clinical failure, (≥ 1 of the following: extension of original antibiotic course, unplanned surgical intervention related to SEA, recurrence of *S. aureus* bacteremia, and all-cause mortality) by OPT. Secondary outcomes included components of the primary outcome and composite 90-day clinical failure by methicillin susceptibility and EPT.

**Figure 1.**
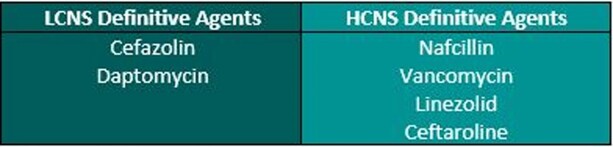
Lower (LCNS) vs Higher (HCNS) CNS Penetration Agents for Definitive Therapy

**Results:**

174 patients met inclusion: 94 (54%) in the LCNS group and 80 (46%) in the HCNS group. There was no significant difference in 90-day clinical failure of OPT between groups (Fig. 2). The composite 90-day clinical failure of OPT also did not differ between LCNS and HCNS based on methicillin susceptibility (MSSA 25.9% vs. 19.2%, p = 0.49; MRSA 30.8% vs. 31.5%, p = 0.96). Similarly, there was no difference in 90-day clinical failure based on EPT (31% vs. 24%, p = 0.28), regardless of methicillin susceptibility, (MSSA 29% vs. 17.8%, p = 0.18; MRSA 50% vs. 28.8%, p = 0.23) between the LCNS and HCNS groups, respectively (Fig. 3).

**Table 1.**
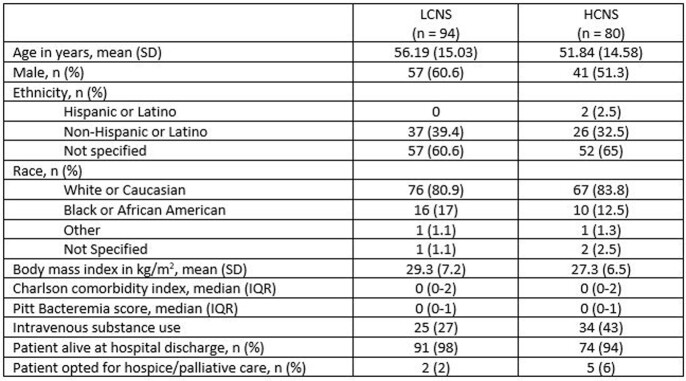
Selected Baseline Characteristics

**Figure 2**

**Figure:**
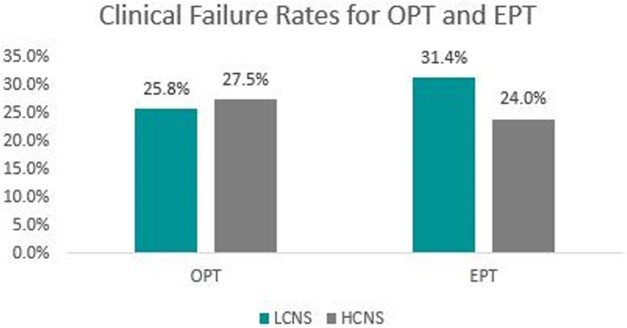

**Figure 3**

**Figure:**
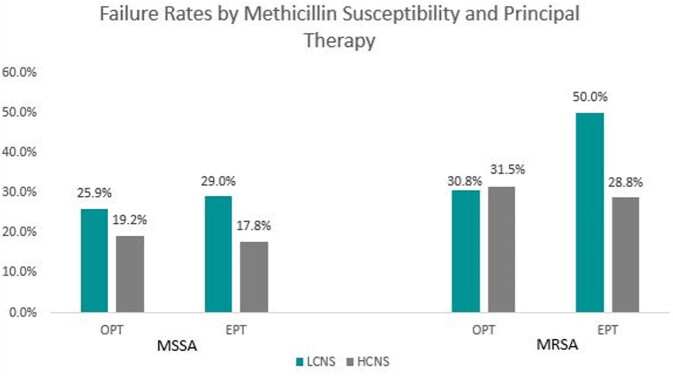

**Conclusion:**

There was no significant difference in the rate of 90-day composite clinical failure between OPT with LCNS and HCNS. Given the limited sample size, further study, particularly in an MSSA SEA cohort, is warranted.

**Disclosures:**

**All Authors**: No reported disclosures

